# Massive Hiatal Hernia With Acute Gastric Volvulus Masked as a Suspected Food Poisoning: A Case Report

**DOI:** 10.7759/cureus.44943

**Published:** 2023-09-09

**Authors:** Landon McNellage, Zachary S Pacheco, Erin F Shufflebarger

**Affiliations:** 1 College of Medicine, University of Alabama at Birmingham School of Medicine, Birmingham, USA; 2 Emergency Medicine, University of Alabama at Birmingham School of Medicine, Birmingham, USA

**Keywords:** hiatal hernia, abdominal surgical emergency, emergency abdominal surgery, acute abdominal pain, gastric volvulus

## Abstract

We describe the case of a 69-year-old female who thought she had "food poisoning", which prompted her visit to the Emergency Department for evaluation of her vomiting and abdominal discomfort. Contrasted computed tomography imaging with contrast of the abdomen subsequently revealed the diagnosis of gastric volvulus, and the patient was promptly taken for surgical intervention. Gastric volvulus is rare and presents with a nonspecific history, exam, and laboratory findings. In the acute care setting, it is important to maintain a high clinical suspicion for this diagnosis, as timely imaging and intervention are crucial to decreasing patient morbidity and mortality.

## Introduction

For a patient with gastric volvulus, or rotation of the stomach along one of its axes, early diagnosis and treatment are crucial to preventing mortality [1]. Gastric volvulus is rare, and its clinical presentation varies, making it difficult to diagnose [2]. Many nonspecific symptoms, such as abdominal pain, vomiting, gastrointestinal bleeding, and anemia, have been identified in a study examining all patients presenting with acute volvulus over a 10-year period [3]. This case highlights the non-specific symptoms of a patient ultimately diagnosed with gastric volvulus and how emergency medicine or other acute care providers can play a role in recognition, timely imaging, and gastric decompression, leading to better patient outcomes.

## Case presentation

A 69-year-old female with a history of laparoscopic appendectomy 15 years ago but no other medical history presented to the emergency department (ED) with nausea and vomiting. The patient reported multiple episodes of emesis since symptom onset the evening before presentation. She denied hematemesis, diarrhea, and fever. The patient denied any significant abdominal pain, only reporting that the episodes of straining during emesis led to her abdominal muscles feeling constantly sore.

On a physical exam, she was alert and oriented but appeared to be in moderate distress. She was afebrile at 97.6° F, her heart rate was 97 beats per minute, her oxygen saturation on pulse oximetry was 96%, and her blood pressure was 189/115 mm/Hg. Her cardiopulmonary exam was unremarkable, and she had normal distal peripheral perfusion. Her abdomen was soft and non-distended, with mild generalized tenderness to palpation but no rebound or guarding.

**Figure 1:**
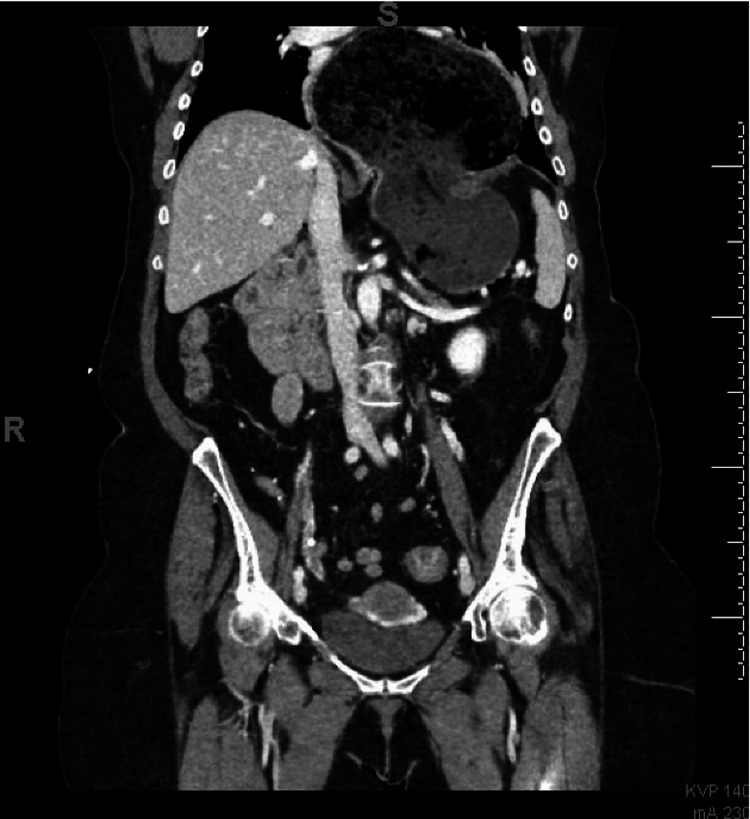
A computed tomography scan of the abdomen and pelvis with contrast revealed hiatal hernia with gastric volvulus and obstruction at the level of the diaphragm.

Laboratory testing revealed mild hyperglycemia at 207 mg/dL and leukocytosis at 20.86x103 with 93% neutrophils. Otherwise, electrolytes were within normal limits. Considering the patient's age and nonspecific presentation, CT imaging of the abdomen and pelvis with contrast was obtained to further evaluate her generalized abdominal tenderness. CT (Figure 1) revealed a massive hiatal hernia with organoaxial volvulus resulting in gastric obstruction at the level of the diaphragmatic hiatus, with evidence of microperforation and possible ischemia of the significantly distended portions of the stomach present within the hiatal hernia. 

Following CT imaging, the general surgery team on call was consulted, and a nasogastric (NG) tube was placed with an immediate output of 500 cc of gastric contents. Less than three hours after the CT scan was obtained, the patient was promptly taken to the operating room for esophagogastroduodenoscopy (EGD). The EGD revealed gastric volvulus at the pylorus/duodenum in the intrathoracic portion of the hernia, with patchy mucosal necrosis observed. She underwent endoscopic gastric decompression and was admitted after this procedure, with plans for subsequent hiatal hernia repair during her hospital stay. Five days after her initial ED presentation and EGD, she underwent laparoscopic hiatal hernia repair and gastropexy. She was discharged home after a seven-day hospitalization without any significant postoperative complications. At the time of discharge, her pain was controlled, and she was tolerating a regular diet with the return of normal bowel function.

## Discussion

This case describes a patient with a rare condition that can easily be misdiagnosed. Maintaining a high level of suspicion and obtaining prompt imaging is crucial to identifying the presence of gastric volvulus, as there are no specific history, or exam findings or diagnostic lab values. Although, previously barium swallow was the gold standard for diagnosis of gastric volvulus, CT scans are most accurate at diagnosing acute gastric volvulus [4]. Gastric volvulus can be the result of primary or secondary causes. Primary causes include defects or abnormalities in the absence of a diaphragmatic hernia, such as gastric ligament elongation or agenesis. More commonly, secondary gastric volvulus is a result of other anatomic abnormalities, such as a paraoesophageal or diaphragmatic hernia [5]. This patient presents with a case of secondary gastric volvulus due to the presence of a diaphragmatic hernia. Preferred management of gastric volvulus due to hernia includes endoscopic reduction of the volvulus and subsequent laparoscopic hernia repair [6].

Regarding the ED provider’s role in the diagnosis and management of gastric volvulus, a few things should be considered. Due to the nonspecific symptoms and lab values, a high index of suspicion must be present, specifically in patients who are greater than 50 years old, given the increased risk of gastric volvulus in this age group [7,8]. CT imaging of the abdomen should be considered for patients with acute abdominal symptoms in the appropriate age group and is the most accurate way to diagnose gastric volvulus. While the risks of CT imaging should be weighed, prior literature has demonstrated that patients greater than 60 years old presenting with abdominal pain are more likely to have an atypical presentation of disease and have an increased risk of serious disease. Therefore, CT imaging should be strongly considered in this high-risk population [9]. Furthermore, prompt imaging and diagnosis lead to decreased morbidity and mortality in patients with acute gastric volvulus, specifically in cases of strangulation [7]. Once a diagnosis has been made, timely gastric decompression, beginning with the placement of a NG tube, enables improved perfusion and decreases overall gastric tension while awaiting definitive operative repair. This is imperative in patients with acute gastric volvulus, as untreated volvulus leads to ischemia and necrosis [1,4-5] and has a mortality rate of 30%-50% [7].

Although definitive treatment in patients with acute gastric volvulus is surgical intervention, the emergency department providers’ decisions to order a CT scan in the older adult patient presenting with acute, non-specific abdominal symptoms could lead to the correct diagnosis of gastric volvulus [7-9]. Furthermore, placing an NG tube for initial gastric decompression immediately after the diagnosis could enable better patient outcomes before definitive surgical reduction and repair. 

## Conclusions

It is important that emergency physicians are aware of this rare diagnosis and consider it when evaluating patients with relevant histories and exam findings, as timely diagnosis and management are important for reducing morbidity and mortality.
